# Factors Associated with Pre-Dental Students’ Intention and Willingness to Serve in the Underserved Community and Vulnerable Population

**DOI:** 10.3390/dj10060111

**Published:** 2022-06-14

**Authors:** Brent Lin, Jungsoo Kim, Michael Lin, Jyu-Lin Chen

**Affiliations:** 1 Division of Pediatric Dentistry, School of Dentistry, University of California, San Francisco, CA 94118, USA; jungsoo.kim@ucsf.edu; 2 Department of Psychiatry, University of Texas Southwestern Medical Center, Dallas, TX 75390, USA; dr_michael_lin@yahoo.com; 3 Department of Family Health Care Nursing, School of Nursing, University of California, San Francisco, CA 94118, USA; jyu-lin.chen@ucsf.edu

**Keywords:** underserved community, vulnerable population, pre-dental students, career in dentistry, underrepresented minority, children, career path, predictors

## Abstract

A potential solution to the problem of how to increase access to dental care for the underserved and vulnerable populations is to establish an early pipeline of underrepresented and minority college students for a career in dentistry. This study aims to explore factors associated with such pre-dental students’ future intentions to serve. A cross-sectional design was utilized with 144 participants completing the questionnaire with four sections, including participants’ demographics, experience in access to dental care, psychosocial factors, and intention to serve the underserved and vulnerable populations. Descriptive statistics, chi-squared test, and logistic regression were used for statistical analyses. A positive attitude (OR = 12.03) and higher confidence towards addressing access to dental care issues (OR = 10.43) were found to be the strongest factor for higher intention to serve the underserved and vulnerable populations. Higher knowledge on the prevalence of dental caries among children (OR = 3.18) and participants who experienced difficulty in getting a dental appointment, or finding an available dentist when needed (OR = 3.43), were also associated with higher intention. Identifying key factors associated with higher intention to serve the underserved and vulnerable populations as a future dentist may facilitate workforce recruitment in the Health Profession Shortage Areas (HPSAs).

## 1. Introduction

Dental caries is the most common chronic disease in childhood [1], and, similar to infectious diseases, the pathogenic transmission is preventable and the course of invasiveness could be deterred or reversed through early detection and timely intervention [2]. Serious consequences have resulted from untreated dental decay as children suffer from toothache, compromised nutritional intakes, increased school absenteeism, poor academic performance, decreased self-esteem, and increased visits to the emergency room [3]. Strong and significant correlations were found between caries experience and dental pain (Spearman’s coefficient 0.86 to 0.95) among children in the low socioeconomic stratum and experiencing reduced access to care [4]. Another study reported a significantly higher caries incidence (*p* < 0.01) in children with immigrant backgrounds compared to non-immigrant young children under 3.5 years of age [5]. Similarly, children in the under-represented or disadvantaged groups, and those with special needs, also suffered from a disproportionately high incidence of dental disease and untreated dental caries [6]. Despite efforts among dental professionals in disease prevention and measures to reduce dental caries, caries prevalence has remained high in certain populations, especially among those in the underserved and rural communities, as well as in the vulnerable population [7].

Lack of access to dental care has been associated with increased oral health disparities among children; and geographic, cultural, and economic factors have been identified as potential barriers to access to dental care [8]. Families may have difficulty accessing care due to a lack of resources, oral healthcare providers, or dentists participating in the state welfare program. Rural and remote areas are known to have a shortage of healthcare providers, and those severely affected areas have been designated as Health Profession Shortage Areas (HPSAs). The shortage of primary care providers poses a significant issue on access to care in rural and remote areas [9].

Deficiency in health literacy and oral health education could have an adverse impact and undermine the critical role of health on children’s wellbeing [10]. Communities tend to foster a trusting relationship with healthcare providers who speak their language, understand their culture, align with their beliefs, and come from a similar ethnic background. Language and cultural barriers may hinder families from receiving appropriate health information and prevent individuals from seeking routine dental care and periodic oral evaluation. Language difficulty may lead to miscommunication, treatment dissatisfaction, and inefficient or inadequate care. In a study by Tang et al., linguistic minorities encountered more medical errors and received worse care with more adverse outcomes than English-speaking patients [11]. Effective communication can ease relations between patients and care providers, increase health literacy, and promote higher acceptance of proposed treatment and care recommendations.

Studies have also shown that more collaborative communication is associated with better adherence to taking medication in both race-discordant relationships and race-concordant relationships between patients and healthcare providers. However, race-concordant patient-provider relationships resulted in more positive effects, satisfaction, and longer medical visits [12]. Hence, diversity in the health care workforce is critical as it affects care quality, outcomes, satisfaction with care, enhanced provider-patient communication, safety, and fosters trusting relationships between patients and providers. A non-diverse workforce could lead to bias, cultural and linguistic barriers, and compromised relationships and clinical outcomes [13].

To address access to dental care and the barriers, a potential solution is to recruit oral healthcare providers from the underserved communities, establish an early pipeline of underrepresented and minority college students for a profession and career in dentistry, and therefore increase the dental workforce, when these practitioners return and serve in underserved communities. Brown and colleagues found that healthcare providers from economically disadvantaged and minority backgrounds are more likely to practice in the underserved areas with a large underrepresented population, and serve and take care of patients and communities with similar backgrounds [14]. Studies have also shown that African American, Hispanic American, and Native American healthcare providers are more likely to practice in underserved communities, serve minority patients, and provide care for those on Medicaid or in low socioeconomic status [15,16]. However, the number of African American and Hispanic American healthcare trainees and providers is disproportionately lower among all healthcare trainees and providers in the U.S. [17]. Underrepresented minority students may not have the same opportunity and access to guidance on healthcare career choice compared to their non-minority peers [18]. One study has also shown that first-generation students from low socioeconomic households tended to have difficulties in the classroom and their neighborhood due to economic, familiar, and socioemotional stressors [19]. The resulting inadequacy in, and insufficient number of, minority and underrepresented primary care providers could be a cause for concern regarding the future distribution of healthcare providers to meet the needs of vulnerable populations in the underserved community [20]. 

To proactively establish an early pipeline for future oral healthcare providers in the underserved, vulnerable, and rural communities, a full-day activity was created to provide an opportunity to learn about the dental profession as a career and elicit interest in joining the dental workforce, through interactive educational activities, career advice, experience on simulated dental procedures, interaction with current dental students, and mentorship for invited college students from Dental HPSA, under-represented minorities, or from disadvantaged backgrounds. These college students were introduced to dentistry as a profession with emphasis on serving the communities and motivational discussion to let the student participants be aware that they have the power to make a difference in communities and address critical health care needs in the designated HPSA. Students are a part of an annual cohort to participate in activities designed to provide an in-depth understanding of dentistry as a career, with the incorporation of elements such as the culture of the profession and steps to accomplish career goals in health care. Current dental students, who participated as cohorts in previous years, shared their successful experiences, and peer support was strategically placed in the activity design. It is critical to increase the pipeline of diverse healthcare providers and reduce disparities [21]. 

This study aims to explore participant characteristics, participants’ past experiences in receiving dental care and to identify factors, including psychosocial (knowledge, confidence, attitude, and past behavior), associated with participants’ higher intention to serve the underserved and vulnerable populations as a future dentist.

## 2. Materials and Methods

This study utilized a cross-sectional design to identify factors associated with the intention to serve the underserved and vulnerable populations among participants interested in a dental health career. This study has been approved by the University of California, San Francisco’s (UCSF) Institutional Review Board (16-19360).

This study was conducted at an annual pre-dental outreach event hosted by an inter-professional health group focused on addressing issues on oral health disparity and access to care issues. The event was held at UCSF in Northern California, and participants were recruited from 2016, 2017, 2018, and 2019 events. All potential participants were approached by the study investigators, and those participants who were willing and consented to participate completed the Qualtrics survey electronically at the beginning of the event. The method of subject recruitment was convenience sampling, and all the participants invited to participate in this study agreed, consented, and completed the survey.

***Questionnaire:*** The questionnaire was comprised of four sections, including demographics, past experience in access to dental care, psychosocial factors, and the intention to serve underserved and vulnerable populations:

1. **Demographics and Characteristics:** Participants’ age, gender, race, ethnicity, yearly family income, current educational status, undergraduate institution, undergraduate study major, and whether they were first-generation college students, and those from an underrepresented minority, or from disadvantaged or rural backgrounds were included.
2. **Past Experience in Access to Dental Care (4 questions):** Participants were asked whether they had a previous difficult experience in getting a dental appointment or finding an available dentist when needed, financial difficulty in affording dental care, transportation difficulty in getting to a dental office, and whether they had experienced difficulty in receiving dental care due to language barriers. Answer choices were “Yes” (1 point) or “No” (0 points).
3. **Psychosocial Factors (Table 1):**
  - *Knowledge (1 question):* Participants were asked how much they agree with the statement “Dental caries is the most common chronic disease in children.” Answer choices were Strongly Agree, Agree, Disagree, and Strongly Disagree. The responses were then dichotomized and scored as “Strongly Agree” (1 point) and “Less than Strongly Agree” (0 points). Participants who answered “Strongly Agree” were considered as having higher knowledge of the prevalence of dental caries among children than those who had “Less than Strongly Agree.”
  - *Confidence (1 question):* Participants were asked how much they agree with the statement, “I believe I can make an impact in reducing oral health disparities in my community.” Answer choices were Strongly Agree, Agree, Disagree and Strongly Disagree. The responses were then dichotomized and scored as “Strongly Agree” (1 point) and “Less than Strongly Agree” (0 points). Participants who answered “Strongly Agree” were considered to have higher confidence in making an impact in the community compared to those who had “Less than Strongly Agree.”
  - *Attitude (2 questions):* Participants were asked how important it is for them to address these issues as future dentists: “Access of dental care in underserved, vulnerable and rural communities” and “Access to dental care for children.” Answer choices were Very Important, Important, Slightly Important, and Not Important. Participants who answered “Very Important” for both questions (1 point) were considered as having a higher attitude towards addressing access to dental care issues compared to those who had “Less than Very Important” (0 points).
  - *Previous Volunteer Behavior (2 questions):* Participants were asked when they had the opportunity in the past, how often they had “Volunteered for an underserved community” and “Volunteered for children.” Answer choices were Always, Often, Sometimes, Never. Participants who answered “Never or Sometimes” for both questions were considered having lower volunteer behavior (0 points), and those who answered “Always or Often” for at least one question were considered having a higher volunteer behavior (1 point).
4. **Intention to Serve the Underserved and Vulnerable Populations (4 questions):** Participants were asked, when they become dentists in the future, how likely are they do the following: “Serve children,” “Serve vulnerable populations,” “Serve underserved populations,” and “Practice in underserved communities.” Answer choices were Very Likely, Likely, Unlikely, and Very Unlikely. Participants who answered “Very Likely” to all 4 questions were considered to have a higher intention to serve the underserved and vulnerable populations (1 point) compared to those who answered “Less than Very Likely” (0 points).

***Statistical Analysis:*** Descriptive statistics were used to summarize participants’ demographics and characteristics. A chi-squared test was used for identifying the association between each factor and intention to serve the underserved and vulnerable populations. Logistic regression with forwarding selection, odds ratio, and 95% confidence interval was used for multivariate analyses to identify factors associated with intention to serve the underserved and vulnerable populations. Model 1 included all variables in the participant characteristics. Model 2 included participant characteristics and past experience in receiving dental care. Model 3 included participant characteristics, past experience in receiving dental care, and psychosocial factors. SPSS 27 was used for data analysis, and the result is statistically significant at *p* < 0.05. 

## 3. Results

**Participant Characteristics:** A total of 144 participants were recruited, and all 144 participants consented to participate in the study. The participation rate was 100%. Participants (N = 144) were: 70% between 20–29 years old, 70% female, 46% Asian, 20% Hispanic or Latino Ethnicity, 55% yearly family incomes <$50,000, 62% current college student, 62% first-generation college students, 62% underrepresented minority, 51% from disadvantaged background, and 15% from rural residential background (Table 2).

**Unique factors Associated with Participants’ Intention to Serve the Underserved and Vulnerable Populations as a Future Dentist based on Chi-Square analysis:**

1. Participant Characteristics: Participants from underrepresented minority, disadvantaged background, rural residential background were all significantly associated with higher intention to serve the underserved and vulnerable populations (*p* = 0.006, 0.015, 0.013, respectively) (Table 3). Although not statistically significant, first-generation college students also reported higher intention to serve such populations with nearing significance (*p* = 0.057). Regression analyses revealed no statistical difference in the outcome by gender or race.
2. Past Experience in Receiving Dental Care: Having difficulty in getting a dental appointment or finding an available dentist when needed was significantly associated with higher intention to serve the underserved and vulnerable populations in the future (*p* < 0.001) (Table 3). Previous experience of having difficulty in receiving dental care due to financial reasons, transportation to the dental office and language barriers were also associated with higher intention to serve the underserved and vulnerable populations as a future dentist (*p* = 0.005, 0.007, 0.003 respectively).
3. Psychosocial Factors: Participants’ knowledge on the prevalence of dental caries among children, confidence in making an impact in the community, attitude towards addressing access to dental care issues, and previous volunteer frequency were all highly correlated with higher intention to serve the underserved and vulnerable populations as a future dentist (all *p* < 0.001) (Table 3).

**Factors associated with Participants’ Higher Intention to Serve the Underserved and Vulnerable Populations as a Future Dentist using logistic regression (Table 4):**

1. Participant Characteristics (Model 1): Participants from a rural residential background, compared to non-rural residential background participants, were 1.7 times more likely to have higher intention to serve such populations (OR = 1.73, 95% CI = 1.1–28.66). The model has an adequate overall goodness of fit (Hosmer-Lemeshow test, Chi-square = 3.45, *p* = 0.90) with Nagelkerke R square of 0.15.
2. Participant Characteristics + Past Experience in Receiving Dental Care (Model 2): Participants who have past experience of difficulty in getting a dental appointment or finding an available dentist when needed were found to be a strong predictor for having higher intention to serve the underserved and vulnerable populations (OR = 1.45, 95% CI = 1.22–14.83). The model presents a good overall goodness of fit (Hosmer-Lemeshow test, Chi-square = 2.87, *p* = 0.94) with Nagelkerke R square of 0.28.
3. Participant Characteristics + Past Experience in Receiving Dental Care + Psychosocial Factors (Model 3): Higher attitude towards addressing access to dental care issues and higher confidence in making an impact in the community were found to be the strongest predictors for higher intention to serve the underserved and vulnerable populations (OR = 2.78, 95% CI = 2.60–98.96 and OR = 2.60, 95% CI = 1.24–147.47 respectively). Higher knowledge on the prevalence of dental caries among children was also a predictor of higher intention (OR = 1.34, 95% CI = 1.04–13.89). The model has an adequate overall goodness of fit (Hosmer-Lemeshow test, Chi-square = 4.91, *p* = 0.77) with Nagelkerke R square of 0.53.

## 4. Discussion

This study is one of the first studies to identify factors associated with intention to serve underserved and vulnerable populations among participants interested in becoming dental health care providers. We investigated participants’ characteristics, past personal experience in receiving dental care, psychosocial factors, and their association with the intention of serving underserved communities and vulnerable populations when a member of the future dental workforce. Positive attitude, confidence, knowledge, and experience in access to dental care are vital factors associated with a higher intention to serve the underserved and vulnerable populations as a future dentist. Data and outcome of the study may facilitate and strategize a potential solution to resolving the shortage of dental workforce in Health Profession Shortage Areas (HPSAs) and to decrease health disparities in these areas and among vulnerable populations. 

A cross-sectional survey over four years with the majority of participants from a low socioeconomic background, and in underrepresented minority groups, was conducted. The study participants’ demographic data were consistent with the national trend as more women participated and showed interest in the dental profession as a career than men [22]. More than half of the cohort (62%) were first-generation college students. Recruiting students from diverse and less privileged backgrounds is essential to developing a workforce that mimics the population and increases dental health among vulnerable populations.

We found that participants from underrepresented minority groups, disadvantaged backgrounds, or rural residential backgrounds have significantly higher intention to serve the underserved community and the vulnerable population in the future than those who are not from underrepresented minority groups, disadvantaged backgrounds, or rural residential backgrounds. Although not statically significant, a positive trend is associated with first-generation college students serving underserved communities and vulnerable populations. Similar to Brown’s study, individuals or healthcare providers from economically disadvantaged and minority backgrounds are more likely to practice in underserved areas and care for patients in communities with similar backgrounds [14]. To address the healthcare workforce shortage, a strategic plan was developed by the U.S. Department of Health and Human Services and reported in 2021 [23]. Under Health Workforce Strategic Plan CARES ACT Section 3402, Objective 1.2 lays out strategies and plans to increase and achieve diversity, inclusion, and representations in the health professions. Factors associated with demographics and characteristics in the recruitment of future healthcare providers are one of the focal points within the objective. Specifically, the goals are to provide training assistance to individuals with low incomes and from disadvantaged backgrounds, promote opportunities for advancement, actively recruit, train, and retain students from underrepresented backgrounds, and conduct targeted recruitment of American Indian and Alaska Native individuals. Financial incentives would be considered for individuals who otherwise could not afford to pursue education, training, and a career in healthcare [23].

Among study participants, those who have personally experienced problems with access to dental care have a higher intention to, and are more likely to, serve the underserved community and vulnerable population. The correlation between personal experience and participants’ intention to serve those in need perhaps created the driving force for these participants to address oral health disparity and inequality. Issues on the access of dental care, whether due to barriers or obstacles in finding dental care providers in the proximity, finance, transportation, or language barriers, have a tremendous negative impact on these future healthcare providers leading to their willingness to commit and to dedicate their careers to serving the underserved and vulnerable populations. 

It appeared that increasing knowledge and understanding of the prevalence of dental problems among children in the underserved community has a profound impact and significant positive correlation in the intention to serve in Health Profession Shortage Areas. Those participants who have the confidence to reduce oral health disparities are more likely to step forward and accept the challenges of serving the underserved and vulnerable populations. In the same token, those study subjects who believe that it is very important for them to address access to dental care for children and in the underserved, vulnerable, and rural communities as future dentists have higher intention to work with and serve such communities. 

Previous experience working with children and in the underserved communities positively correlates to the willingness and intention to serve in the underserved communities and vulnerable populations. Compared to participants with limited experience, those who frequently volunteered in those communities in need and worked with vulnerable populations, including children, are more likely to practice and provide patient care in a similar setting, and the correlation is significant. A study has shown that work experience while in school, whether voluntary or paid, opened individuals’ eyes to career possibilities and became career concentration after leaving school [24].

To identify the factors associated with intention to serve the underserved and vulnerable populations, the study utilized three statistical models and found that participants from a disadvantaged background are twice as likely to express the intent to serve the underserved and vulnerable populations, compared to their peers from non-disadvantaged backgrounds, while the intent to serve for participants living in rural areas is five times more than those living in non-rural residential areas. The result is similar to a study on healthcare providers using the data from the California Medical Board Physician Licensure Survey [25]. Underrepresented minority physicians were more likely to work in medically underserved areas and health professional shortage areas. Hence, recruiting healthcare providers from disadvantaged communities is critical in addressing the workforce issue in such communities. Other important factors associated with higher intent to serve the underserved areas and vulnerable populations are the participants’ own personal experience on the difficulty in accessing dental care and their knowledge or understanding of the extent and severity of dental disease manifestation in children. However, the strongest predictor to serve the underserved community and vulnerable population, with an odds ratio of 12, is the participant’s attitude and confidence in their ability to address the critical issue of dental care access and make an impact in the community. Our results are consistent with other studies indicating the importance of curriculum focusing on knowledge, attitude, and confidence in one’s ability to make changes are key factors associated with behaviors and intention to provide health care services in the underserved populations [26,27].

The authors acknowledge that the cross-sectional design may possess limitations in the study. A longitudinal study with a follow-up survey after completion of a professional degree and beginning of a career could more precisely track accuracy and verify the extent of the existing data. Although such data is currently unavailable, cases of success among participants in the cohort have been reported. For instance, an underrepresented minority participant in the 2017 cohort group entered dental school in 2018. Upon successful completion of professional education, the participant entered a post-graduate specialty program, as there is a shortage of dental specialists in the underserved community. The intent was to return to serve in such a community and address the need.

The study presented cross-sectional data and analysis that could potentially strategize and develop plans in addressing the dental workforce shortage in the future. Several factors associated with serving in the underserved and vulnerable populations have been identified. 

## 5. Conclusions

1. Participants’ demographics and characteristics are strong determinants of future intent to serve underserved communities and vulnerable populations.
2. Personal experiences of difficulties in dental care access and past volunteering experience in underserved communities and working with vulnerable populations have a significant influence on participants’ intent to serve such populations and communities.
3. Attitude, confidence, and knowledge appeared to be strong predictors among participants’ future intent.

## Figures and Tables

**Table 1:** Questions Related to Knowledge, Confidence, Attitude and Previous Volunteer Behavior.

Questions	Answer Choices
*Knowledge:* 1. How much do you agree with the statement “Dental caries is the most common chronic disease in children”?	Strongly Agree, Agree, Disagree and Strongly Disagree
*Confidence:* 1. How much do you agree with the statement “I believe I can make an impact in reducing oral health disparities in my community”?	Strongly Agree, Agree, Disagree and Strongly Disagree
*Attitude:* How important it is for you to address these issues as a future dentist? 1. Access to dental care in underserved, vulnerable and rural communities. 2. Access to dental care for children.	Very Important, Important, Slightly Important, Not Important
*Previous Volunteer Behavior:* When you had the opportunity in the past, how often have you volunteered for these causes? 1. Volunteered for an underserved community. 2. Volunteered for children.	Always, Often, Sometimes, Never
*Intention to Serve the Underserved and Vulnerable Populations:* How likely are you to do the following after you become a dentist? 1. Serve children. 2. Serve vulnerable populations. 3. Serve underserved populations. 4. Practice in underserved communities.	Very Likely, Likely, Unlikely, Very Unlikely

**Table 2:** Summary of Participant Demographics and Characteristics.

Demographics and Characteristics	Frequency N = 144 n (%)
Age (in years)	
• 19 and under	35 (25%)
• 20–29	97 (70%)
• 30 and above	6 (5%)
Gender	
• Female	97 (70%)
• Male	41 (30%)
Race	
• Asian	64 (46%)
• Caucasian	33 (24%)
• Hispanic or Latino	23 (17%)
• African American	5 (4%)
• Native Hawaiian/Pacific Islander	3 (2%)
• Other	10 (7%)
Ethnicity	
• Not Hispanic or Latino	109 (80%)
• Hispanic or Latino	27 (20%)
Family Yearly Income	
• <$10,000	18 (13%)
• $10,000–$50,000	57 (42%)
• >$50,000	61 (45%)
Current Status	
• College Student	84 (62%)
• Enrolled in Post-Baccalaureate Program	34 (25%)
• Recent College Graduate/Gap Year	18 (13%)
College Institution Enrolled/Attended	
• University of California	58 (44%)
• California State University/College	56 (42%)
• Community College	9 (7%)
• Private University/College	6 (4%)
• Other	5 (4%)
Study Major	
• Science	111 (84%)
• Social Sciences	7 (5%)
• Other	15 (11%)
First-Generation College Student	85 (62%)
Underrepresented Minority	84 (62%)
Disadvantaged Background	51 (51%)
Rural Residential Background	15 (15%)

**Table 3:** Factors Associated with Participants’ Intention to Serve the Underserved and Vulnerable Populations.

	Higher Intent N = 79	Lower Intent N = 62	*p*
Gender (n = 135)			0.5
• Male	21 (28%)	20 (33.3%)	
• Female	54 (72%)	40 (66.7%)	
Race (n = 135)			0.93
• White	18 (24%)	14 (23.3%)	
• Non-White	57 (76%)	46 (76.7%)	
Underrepresented Minority (n = 133)			0.006
• Yes	55 (73%)	29 (50%)	
• No	20 (27%)	29 (50%)	
Disadvantaged Background (n = 98)			0.015
• Yes	35 (61%)	15 (37%)	
• No	22 (39%)	26 (63%)	
Rural Residential Background (n = 99)			0.013
• Yes	13 (23%)	2 (5%)	
• No	44 (77%)	40 (95%)	
First-Generation College Student (n = 135)			0.057
• Yes	52 (69%)	32 (53%)	
• No	23 (31%)	28 (47%)	
Experienced difficulty in getting a dental appointment or finding an available dentist when needed in the past (n = 133)			<0.001
• Yes	38 (51%)	9 (15%)	
• No	36 (49%)	50 (85%)	
Experienced financial difficulty in affording dental care in the past (n = 133)			0.005
• Yes	56 (76%)	31 (53%)	
• No	18 (24%)	28 (47%)	
Experienced transportation difficulty in getting to a dental office in the past (n = 133)			0.007
• Yes	20 (27%)	5 (8%)	
• No	54 (73%)	54 (92%)	
Experienced difficulty in receiving dental care due to language barriers in the past (n = 133)			0.003
• Yes	39 (53%)	16 (27%)	
• No	35 (47%)	43 (73%)	
Knowledge (n = 141)			<0.001
• Higher	65 (82%)	29 (47%)	
• Lower	14 (18%)	33 (53%)	
Confidence (n = 141)			<0.001
• Higher	78 (99%)	40 (65%)	
• Lower	1 (1%)	22 (35%)	
Attitude (n = 141)			<0.001
• Higher	76 (96%)	39 (63%)	
• Lower	3 (4%)	23 (37%)	
Previous Volunteer Behavior (n = 141)			<0.001
• More	11 (14%)	27 (44%)	
• Less	68 (86%)	35 (56%)	

**Table 4:** Predictors of Participants’ Higher Intention to Serve the Underserved and Vulnerable Populations.

	Model 1: Participant Characteristics			Model 2: Participant Characteristics + Previous Access to Dental Care			Model 3: Participant Characteristics + Previous Access to Dental Care + Psychosocial Factors		
	OR	95% CI	*p*	OR	95% CI	*p*	OR	95% CI	*p*
Sex (1-Male)									
Race (1 = White)									
Underrepresented Minority (1 = Yes)									
Disadvantaged Background (1 = Yes)									
Rural Residential Background (1 = Yes)	1.73	1.10–28.66	0.040						
First-Generation College Student (1 = Yes)									
Previously experienced difficulty in getting a dental appointment or finding an available dentist when needed (1 = Yes)				1.45	1.22–14.83	0.02			
Previously experienced financial difficulty in affording dental care (1 = Yes)									
Previously experienced transportation difficulty in getting to a dental office (1 = Yes)									
Previously experienced difficulty in receiving dental care due to language barriers (1 = Yes)									
Knowledge (1 = Higher Knowledge)							1.38	1.09–14.6	0.037
Confidence (1 = Higher Confidence)							2.60	1.23–147.47	0.048
Attitude (1 = Higher Attitude)							2.78	2.60–98.96	0.003
Previous Volunteer Behavior (1 = More Often Volunteered)									

## Data Availability

Not applicable.
